# Binder-free and high-loading sulfurized polyacrylonitrile cathode for lithium/sulfur batteries†

**DOI:** 10.1039/d1ra02462k

**Published:** 2021-04-30

**Authors:** Huihun Kim, Changhyeon Kim, Milan K. Sadan, Hyewon Yeo, Kwon-Koo Cho, Ki-Won Kim, Jou-Hyeon Ahn, Hyo-Jun Ahn

**Affiliations:** Department of Materials Engineering and Convergence Technology & RIGET, Gyeongsang National University 501 Jinju-daero Jinju Gyeongnam 52828 Republic of Korea ahj@gnu.ac.kr +82-55-772-2586 +82-55-772-1666; SMLAB 27, Gacheongondan 1-gil, Samnam-myeon, Ulju-gun Ulsan 44953 Republic of Korea

## Abstract

Sulfurized polyacrylonitrile (SPAN) is a promising active material for Li/S batteries owing to its high sulfur utilization and long-term cyclability. However, because SPAN electrodes are synthesized using powder, they require large amounts of electrolyte, conducting agents, and binder, which reduces the practical energy density. Herein, to improve the practical energy density, we fabricated bulk-type SPAN disk cathodes from pressed sulfur and polyacrylonitrile powders using a simple heating process. The SPAN disks could be used directly as cathode materials because their π–π structures provide molecular-level electrical connectivity. In addition, the electrodes had interconnected pores, which improved the mobility of Li^+^ ions by allowing homogeneous adsorption of the electrolyte. The specific capacity of the optimal electrode was very high (517 mA h g_electrode_^−1^). Furthermore, considering the weights of the anode, separator, cathode, and electrolyte, the Li/S cell exhibited a high practical energy density of 250 W h kg^−1^. The areal capacity was also high (8.5 mA h cm^−2^) owing to the high SPAN loading of 16.37 mg cm^−2^. After the introduction of 10 wt% multi-walled carbon nanotubes as a conducting agent, the SPAN disk electrode exhibited excellent cyclability while maintaining a high energy density. This strategy offers a potential candidate for Li/S batteries with high practical energy densities.

## Abstract 2

A simple synthesis procedure to prepare bulk-type SPAN electrodes toward the realization of Li/S batteries with enhanced practical energy densities.

## Graphical abstract

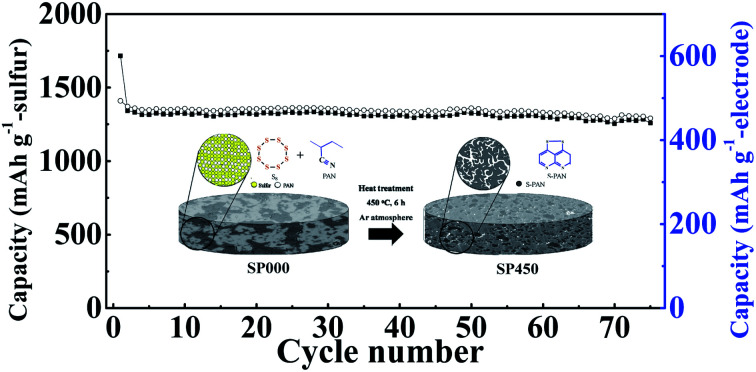

## 1. Introduction

Li/S and Li/O_2_ batteries have potential as next-generation batteries.^1^ Among them, Li/S batteries are widely studied owing to their low cost and high theoretical energy density of 2600 W h kg^−1^.^2,3^ However, Li/S batteries have various drawbacks, such as the insulating properties of sulfur and the high solubility of long-chain lithium polysulfides (Li_2_S_*x*_, 4 ≤ *x* ≤ 8), which are formed from element sulfur during discharge, in ether-based electrolytes such as dimethoxyethane and tetraethylene glycol dimethyl ether.^4–6^ Ongoing efforts have addressed these issues by inserting sulfur into nanostructured carbon^7–9^ or using advanced electrolytes^10,11^ interlayer^12–14^ or a modified separator,^15–17^ or a protected anode.^18–20^ Despite this progress, the practical performance of Li/S batteries remains insufficient to replace lithium-ion batteries.^21^

Current Li/S batteries exhibit low practical energy densities because a low proportion of sulfur in the electrode and large amounts of electrolyte are used to compensate for the insulating properties of sulfur and the solubility of lithium polysulfides, respectively. To achieve high practical energy densities, the following strategies can be adopted: (i) incorporate a minimum amount of inactive materials, such as binders and conducting agents, into the electrode. In particular, conventional sulfur electrodes are generally based on particle-type materials and prepared *via* slurry-casting technology, which uses a polymer binder for adhesion with active materials and a current collector. However, binders have low electrical conductivities, which inhibits electron transfer and reduces the energy density. Therefore, a binder-free, highly interconnected electrode structure with a minimum amount of inactive materials is required. (ii) Introduce synergism between the electrode and electrolyte. Insufficient sulfur loading and the use of excess electrolyte have failed to meet the demands of practical applications. Therefore, to achieve high practical energy densities, sulfur electrodes should be reasonably designed to maintain sufficient electrochemically active sulfur species at a reasonably high sulfur loading with a minimal amount of electrolyte.

As mentioned above, the use of elemental sulfur (S_8_) as an active material is not suitable because large amounts of ether-based electrolytes and conducting agents are required. In previous reports, more than ∼10 μL of electrolyte has typically been used for 1 mg of sulfur.^22–31^ The Chen group reported the use of a minimal amount of electrolyte (∼3 μL of electrolyte for 1 mg of sulfur), but a low specific capacity of 590 mA h g_sulfur_^−1^ was observed, indicating that this amount of electrolyte still reduces the utilization of sulfur.^32^

Another approach is to adopt carbonate-based electrolyte, in which lithium polysulfides are insoluble.^33–35^ Carbonate-based electrolytes can only be used for the ultradispersion of short sulfur chains or small sulfur molecules (S_2_–S_4_) in a carbon matrix such as sulfurized polyacrylonitrile (SPAN)^36–43^ or sulfur-infiltrated specific nanoporous carbon.^44,45^ Unlike S_8_, S_2_–S_4_ can directly form Li_2_S; therefore, when using short-chain sulfur as an active material, the formation of long-chain lithium polysulfides can be ignored.^46^ Consequently, smaller amounts of carbonate-based electrolytes can be used, resulting in enhanced electrochemical performance. Thus, electrically connected bulk SPAN should be synthesized to improve the practical energy densities of Li/S batteries.

Herein, we designed a binder-free and interconnected electrode using a simple compacting and heating process. The obtained bulk-type free-standing sulfur disk electrodes were composed only of SPAN, unlike conventional particle-type electrodes. Therefore, the electrodes had interconnected pores, which improved the Li^+^ ion mobility. In addition, the electrolyte/sulfur ratio was reduced by using a carbonate-based electrolyte with high sulfur utilization (high specific and practical capacities). To determine the optimal preparation conditions for the SPAN disk electrodes, different heating conditions and synthesis methods were investigated. The obtained bulk-type SPAN electrodes exhibited high practical energy densities and good electrochemical performance. In addition, the reaction mechanism of the bulk-type SPAN disk was clarified and the electrochemical properties were improved by adding a minimal amount of conducting agents.

## 2. Experimental

### 2.1. Preparation of bulk-type SPAN disk electrodes

Sulfur (<100 mesh, Sigma-Aldrich) and polyacrylonitrile (PAN; *M*_w_ = 150 000, Sigma-Aldrich) powders were mixed at a weight ratio of 80 : 20 using a mortar and pestle. The mixed powder was compressed by applying a pressure 500 kg in a cylindrical mold with a diameter of 10 mm to form pellets (denoted SP000). The SP000 pellets were covered with Al foil to prevent the evaporation of sulfur at high temperatures. Then, the samples were heat-treated at 150, 300, 450, or 600 °C for 6 h in an argon atmosphere to obtain free-standing bulk-type electrodes (denoted SP150, SP300, SP450, and SP600, respectively). To determine the effect of compression, SP450 was ground using a mortar and pestle. Then, the SP450 powder was compressed under the same conditions, and the resulting sample was denoted SP450compact.

The as-prepared bulk-type SPAN samples were directly used as cathodes without a current collector, conducting agent, or binder. To prepare a conventional sulfur electrode, ground SP450 powder was mixed with multi-walled carbon nanotubes (MWCNTs; CM-95, Hanwha Chemical) and β-cyclodextrin (Sigma-Aldrich) at a weight ratio of 80 : 10 : 10 using distilled water as a dispersant by ball milling at 300 rpm for 3 h. The resulting slurry was coated on Al foil and then dried at 60 °C overnight, and the obtained sample was denoted SPconventional. To improve the performance of SP450, MWCNTs were added to SP000 (sulfur/PAN/MWCNT ratio of 70 : 20 : 10). This mixture was treated under the same conditions used to prepare SP450, and the obtained sample was denoted SP450-C.

### 2.2. Material characterization

The crystalline structures of the electrodes were confirmed by X-ray diffraction (XRD; D8 Advance, Bruker AXS). The thermal decomposition behaviors of the raw materials and the SPpellet samples were determined by thermogravimetric analysis (TGA; Q50, TA instruments). The surface morphologies were examined using field emission scanning electron microscopy (FESEM; JSM-7610F, JEOL) coupled with energy-dispersive X-ray spectroscopy (EDS; X-max, Oxford Instruments). The sulfur contents in the electrodes were determined using elemental analysis (EA; Vario MACRO cube, Elementar). The resistance values of the SPpellet samples were measured using a multimeter inside a glovebox. The molecular structures of the SPpellet samples were identified using Fourier transform infrared (FTIR) absorption spectroscopy (Vertex 80v, Bruker). Detailed characterization of the chemical bonds was performed using an HR micro-Raman spectrometer (LabRAM HR800 UV, Horiba Jobin Yvon) equipped with a 514 nm argon-ion laser. To investigate the changes after charging or discharging, the Li/S cells were disassembled in an argon-filled glovebox and washed three times with diethyl carbonate (DEC) for 5 min to remove lithium salts.

### 2.3. Electrochemical characterization of electrodes

We examined the free-standing SPpellet, SP450compact, and SPconventional samples as sulfur cathodes. Lithium foil (Honjo Metal Co.) and a polypropylene film (Celgard 2400) were used as the anode and separator, respectively. The electrolyte consisted of 1 M LiPF_6_ in ethylene carbonate (EC) and DEC (1 : 1, v/v, Soulbrain Co.). Li/S cells were assembled by sequentially stacking the lithium anode, electrolyte, Celgard 2400 separator, and sulfur cathode. Galvanostatic tests of SP450 were performed at room temperature between 1.0 and 4.0 V *vs.* Li/Li^+^ at a current density of 15 mA g^−1^ based on the electrode weight using a battery cycler (WBCS3000, WonATech Co.). The other cathodes were evaluated between 1.0 and 3.0 V. Cyclic voltammetry (CV) was performed at a scan rate of 1 mV s^−1^ in a voltage rage of 1–3 V. Electrochemical impedance spectroscopy (EIS, VMP3, Bio-Logic) was collected with an amplitude of 10 mV over a frequency range from 10 mHz to 1 MHz for original and cycled cells.

## 3. Results and discussion

**Fig. 1:**
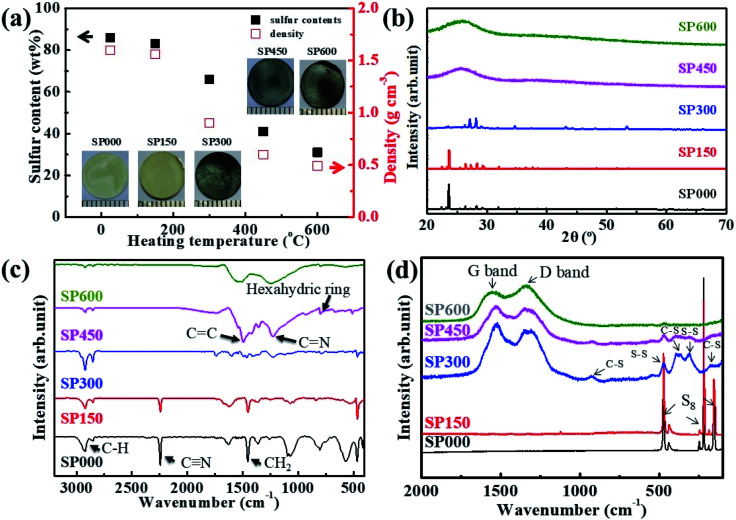
(a) Sulfur contents, densities, and images, (b) XRD patterns, (c) FTIR spectra, and (d) Raman spectra of SPpellet samples.

Although considerable research has already been conducted on SPAN powders, this is the first study of bulk-type SPAN electrodes. The electrode synthesis conditions were optimized by investigating the properties of SPAN electrodes prepared at various heating temperatures (Fig. 1). Fig. 1a shows the apparent densities, sulfur contents, and photographs of the disk-shaped SPpellet samples. Free-standing SP000 is in the form of a yellow disk, corresponding to the typical color of sulfur. The yellow color gradually changed to dark gray as the temperature increased. The apparent density was calculated by considering the volume and mass of the electrode. The apparent density of SP000 was 1.6 g cm^−3^, which is lower than the theoretical density of 1.91 g cm^−3^ (calculated based on the densities of sulfur (2.1 g cm^−3^) and PAN (1.15 g cm^−3^)). This difference could be related to the presence of pores in the pellet.^47^ Initially, the apparent density gradually decreased as the temperature increased and then rapidly decreased from 0.9 g cm^−3^ for SP300 to 0.49 g cm^−3^ for SP600. In addition, the sulfur content of SP000 was similar to that of the precursor mixture (sulfur/PAN ratio of 80 : 20). However, the sulfur contents of the SPpellet samples decreased with increasing heating temperature, with a rapid decrease observed from 65 wt% for SP300 to 30 wt% for SP600. The reduction in the apparent density and sulfur content was due to the sublimation of sulfur. The XRD patterns of the SPpellet samples are shown in Fig. 1b. SP000 exhibits a crystalline peak corresponding to the orthorhombic structure of elemental sulfur. The XRD pattern of SP150 was similar to that of SP000. However, in the case of SP300, the intensity of the orthorhombic peak decreased, indicating a reduction in the amount of elemental sulfur. Neither SP450 nor SP600 exhibited sharp peaks corresponding to elemental sulfur, implying the complete absence of elemental sulfur.

The chemical bonds in the SPpellet samples were further characterized using FTIR and Raman spectroscopy. As shown by the FTIR spectra in Fig. 1c, SP000 had sharp peaks corresponding to C≡N, CH_2_, and CH bonds, which probably originated from PAN ((C_3_H_3_N)_*n*_). The spectrum of SP150 was similar to that of SP000, indicating that no chemical transformation of either PAN or sulfur occurred at 150 °C. In contrast, in the FTIR spectra of the samples prepared above 300 °C, the peaks corresponding to C≡N and CH_2_ bonds disappeared, implying that both cyclization and dehydrogenation started at 300 °C. The cyclization reaction involves the nitrile groups of the PAN precursor. The FTIR spectra of both SP450 and SP600 contained peaks corresponding to C=C and C=N bonds and a hexahydric ring, which are related to the formation of π-conjugated coplanar hexahydric ring structures. As shown in Fig. 1d, the Raman spectrum of SP000 only shows three strong characteristic peaks located at 152, 219, and 472 cm^−2^, which are characteristic of elemental sulfur. The Raman peaks for SP300 appeared at 1325 and 1530 cm^−1^, corresponding to the disorder-induced D band and graphitic G band, respectively. This observation indicates that PAN underwent a structural change to form a carbon structure through dehydrogenation and carbonization. In addition, the Raman spectrum of SP300 contained several small peaks at 475, 387, 305, and 177 cm^−1^, which are characteristic of S–S and C–S bonds. The characteristic peaks in the Raman spectra of SP450 and SP600 were similar to but weaker than those in the spectrum of SP300. The sulfur states of the SPpellet samples were also investigated using TGA, as shown in Fig. S1.† SP000 was stable up to 200 °C. From 200 to 250 °C, SP000 lost ∼80% of its weight owing to the sublimation of S_8_. The TGA curves of SP150 and SP300 were similar to that of SP000. In contrast, SP450 and SP600 were thermally stable to 600 °C. This increased stability is due to the standard bond energy of C–S bonds (740 kJ mol^−1^) being higher than that of S–S bonds (418 kJ mol^−1^).^48^

**Fig. 2:**
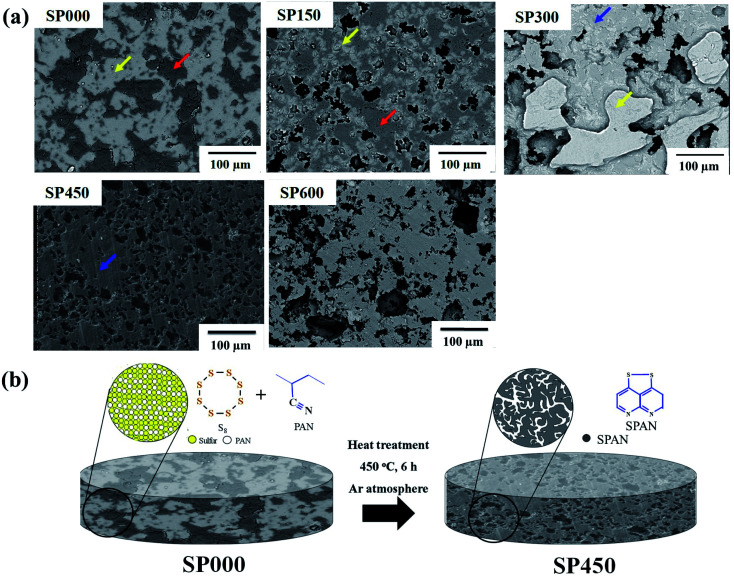
(a) FESEM images of SPpellet samples and (b) schematic diagrams of SP000 and SP450.

SEM images of the SPpellet samples are shown in Fig. 2a. The image of SP000 contains both bright (yellow arrow) and dark gray regions (red arrow). The EDS results (Fig. S2†) revealed that the bright regions contained only sulfur, whereas the dark gray regions contained both carbon and nitrogen. These results clearly indicate that the bright and dark gray regions correspond to S_8_ and PAN ((C_3_H_3_N)_*n*_), respectively. In addition, PAN was surrounded by sulfur because SP000 contained more sulfur than PAN. SP150 had fewer bright regions than SP000 and black regions (red arrow) formed on the SP150 surface, which corresponded to pores formed by sulfur melting at 115 °C. The EDS results showed that the bright gray regions (blue arrow) of SP300 were composed of S, C, and N, indicating the presence of SPAN. The area of SPAN in SP300 was similar to that of PAN in SP150, suggesting the transformation of PAN to SPAN. A bright region corresponding to sulfur remained as a film on the surface near the gray region corresponding to SPAN, which is consistent with the XRD and TGA results (Fig. 1b and S1†). Thus, SP300 has two phases: S_8_ and SPAN. In contrast, SP450 exhibited single-phase SPAN without any S_8_, which also agrees with the XRD and FTIR results. The porosity of the samples is related to the reduction in the density, as shown in Fig. 1a. A high pore volume in an electrode can increase the uptake of the electrolyte. The morphology of SP600 was similar to that of SP450. However, the pore size of SP600 was larger than that of SP450, which may reduce the practical energy density by taking up a large amount of electrolyte. Based on the properties of the SPpellet samples prepared at various heating temperatures, SP450 was directly used as a cathode because in contained only SPAN without any S_8_ (for use with carbonate-based electrolytes) and it had an appropriate amount of pores. However, the synthesis of SPAN particles has been reported at temperatures below 300 °C.^36^ This difference in the optimized heating temperature was due to the electrode structure. Compared with powders, a higher temperature is required for the synthesis of bulk-type SPAN because it is difficult to sublimate sulfur owing to its density. Based on the properties of the SPpellet samples (Fig. 1 and 2a), schematics of the electrode are presented in Fig. 2b. The sulfur and PAN mixture in SP000 was converted to 42.3 wt% SPAN in SP450. The SPAN particles in SP450 were connected to each other owing to the pressing process, which introduces electron-transfer pathways. In addition, the pores in SP450 can take up the electrolyte. The interconnected pores in SP450 can not only serve as electrolyte buffer reservoir to shorten mass diffusion distance but also facilitate fast mass transport and ensure high cycling performance.^49–56^

**Fig. 3:**
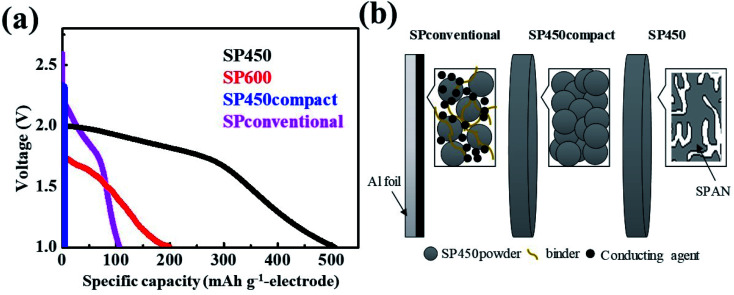
(a) Discharge curves at 15 mA g_electrode_^−1^ and (b) schematic diagrams of various SPAN-based electrodes.

To compare the gravimetric capacities of the electrodes according to the structure and synthesis method (Fig. 3a and S3†), we also synthesized a cast electrode (SPconventional) and a dense electrode (SP450compact). The SPpellet samples prepared at temperature below 300 °C did not exhibit capacity because they contained elemental sulfur, which cannot react in polysulfide-insoluble carbonate-based electrolytes (Fig. S3†). Because the initial cycle was accompanied by an irreversible reaction, the 2^nd^ discharge curves were compared. At the 2^nd^ discharge, the gravimetric capacities of the SP450, SP600, SP450compact, and SPconventional electrodes were 675, 297, 0, and 106 mA h g_electrode_^−1^, respectively. The SP450 electrode exhibited the highest value because it did not contain any inactive components such as a current collector, binder, or conducting agent. The discharge capacity of SP600 was lower than that of SP450 because of its lower sulfur content. Although SPconventional had a high gravimetric capacity of 1931 mA h g_sulfur_^−1^, this electrode exhibited the lowest gravimetric capacity because of the weight of the inactive components (Table S1†). However, it had the highest potential, which may be related to the high electrical conductivity resulting from the conducting agents and the Al current collector. Although SP450 and SP450compact were fabricated using the same materials, the SP450compact electrode exhibited a smaller capacity and a curve with a greater slope than the SP450 electrode (Fig. S3†). Furthermore, the SP450compact electrode could not be charged after the 1^st^ discharge; thus, there is no data for the 2^nd^ discharge. This difference may arise from SP450compact having a high resistance resulting from interfacial resistance within the SP450 powder, whereas SP450 is expected to have an electrical connection path from the bottom to the top of the disk, *i.e.*, continuously interconnected SPAN. To explain these differences in structure, schematic diagrams of the SP450, SP450compact, and SPconventional electrodes are shown in Fig. 3b. SP450 formed an electrically connected structure after heating, allowing this material to be used as a high-gravimetric-capacity free-standing electrode without any inactive components. However, in SP450compact, which was prepared by compressing the prepared SP450 powder, electron transfer was difficult owing to the interfacial resistance of each particle. In contrast, SPconventional contained inactive components such as a binder, conducting agents, and a current collector, which probably contributed to its high electrical conductivity. However, the high ratio of inactive components decreased the gravimetric capacity of the electrode. Although increasing the thickness of the SPconventional electrode would improve the capacity per electrode weight, the electrochemical properties cannot be guaranteed. Moreover, the capacity per electrode weight cannot be increased compared to that of SP450.

**Fig. 4:**
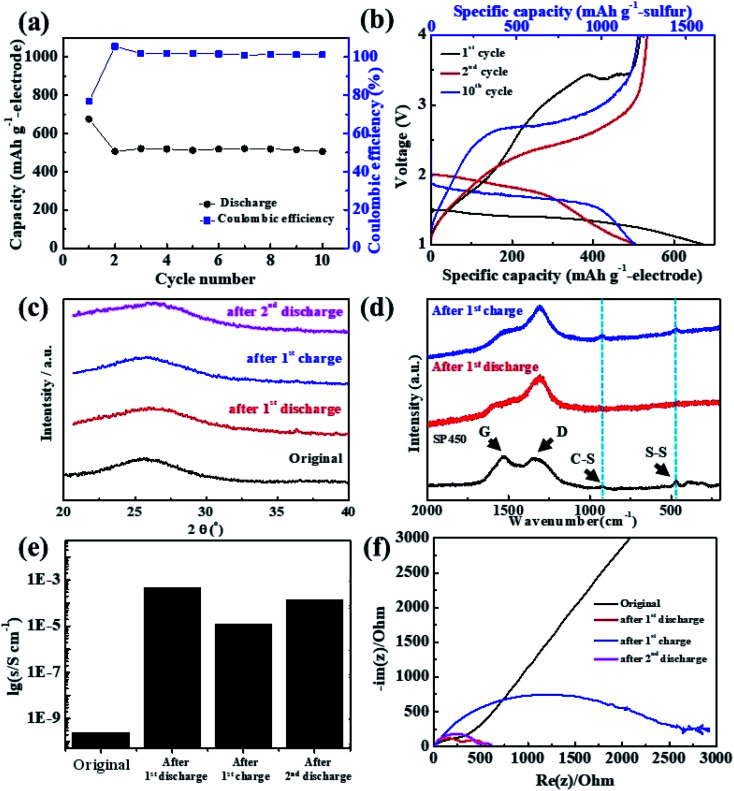
(a) Cyclability and (b) charge–discharge curves of the Li/SP450 cell at current density of 15 mA g_electrode_^−1^. (c) *Ex situ* XRD patterns, (d) *ex situ* Raman spectra, (e) electrical conductivity, and (f) EIS results of the Li/SP450 cell at various steps during cycling.

Owing to the high gravimetric capacity of the SP450 electrode, the performance, the performance of the corresponding Li/S cell was investigated. The cell was constructed using the free-standing SP450 electrode, which was a circular disk with a diameter of 10 mm, thickness of ∼450 μm, and sulfur loading of 6.87 mg cm^−2^. As shown in Fig. 4a, the Li/SP450 cell exhibited stable cyclability at 15 mA g_electrode_^−1^ and maintained a discharge capacity of 517 mA h g_electrode_^−1^ at the 10^th^ cycle, with a coulombic efficiency of nearly 100%. Fig. 4b shows the changes in the charge–discharge curves of the Li/SP450 cell during cycling. The discharge curve had one plateau, the potential of which increased after the 2^nd^ cycle. The Li/SP450 cell exhibited an initial discharge capacity of 674 mA h g_electrode_^−1^, which decreased to 517 mA h g_electrode_^−1^ after the 1^st^ charge. This irreversible behavior may be related to residual lithium in the SPAN matrix.^46^

As SP450 was composed of only SPAN without any inactive components, it can be used to investigate the properties of pure SPAN after cycling. The XRD patterns of SP450 after cycling are shown in Fig. 4c. During lithiation or delithiation, the amorphous structure did not change. Although the crystalline peak of sulfur was not observed, nanocrystalline sulfur species may be present. To obtain a deeper understanding of the reaction mechanism, the bonding characteristics of SP450 during the reaction with Li^+^ ions were further clarified by *ex situ* Raman spectroscopy, as shown in Fig. 4d. Initially, the spectrum of SP450 exhibited characteristic C–S and S–S, which correspond to the sulfur states in SPAN. After full discharge, the C–S and S–S peaks disappeared, indicating that Li_2_S was formed from free sulfur atoms after cleavage of the C–S and S–S bonds. However, the C–S and S–S peaks reappeared after full charge, which means that structural changes involving the formation and cleavage of C–S and S–S bonds were reversible.

The calculated electrical conductivity of SP450 after cycling is shown in Fig. 4e. This is the first report of the electrical conductivity of SPAN after cycling. The initial electrical conductivity of SP450 was 2.45 × 10^−10^ S cm^−1^, which is lower than the previously reported value (5.3 × 10^−4^ S cm^−1^, ∼10^−4^ S cm^−1^),^57,58^ which might be due the SP450 electrode SP450 having a higher porosity than previously reported SPAN-based electrodes. After the 1^st^ discharge, the electrical conductivity of SP450 increased dramatically to 4.66 × 10^−4^ S cm^−1^. Considering the difference in electrical conductivity between the previously reported electrodes and the initial SP450 electrode, discharged SP450 is estimated to have a higher electrical conductivity of ∼10^−2^ S cm^−1^. Furthermore, the electric conductivity was not restored to its initial value after the 1^st^ charge and 2^nd^ discharge. After cycling, SP450 exhibits semiconductor-like electrical conductivity, which is why the cycling performance is good, even without conducting agents or current collectors.

Electrochemical impedance data before and after cycling is shown in Fig. 4f. The Nyquist plots contain a semicircle at high frequencies, which is usually associated with charge transfer, and a sloping straight line at low frequencies, which can be attributed to Li^+^ diffusion into the active materials. The charge-transfer resistance (*R*_ct_) for the pristine electrode was ∼450 Ω, which decreased to 280 Ω after the 1^st^ discharge. However, *R*_ct_ dramatically increased to 2500 Ω after 1^st^ charge and then again decreased to 470 Ω after the 2^nd^ discharge. The initial decrease in *R*_ct_ may originate from the increase in the electrical conductivity of SP450 after the 1^st^ discharge. The subsequent increase in *R*_ct_ may be due to increased resistance during charging. Thus, lithiation can decrease the electrical conductivity and *R*_ct_.

The practical specific energy density is a major factor affecting the performance of Li/S batteries, with the integration of inactive materials decreasing the practical energy density. Critically, the amount of electrolyte can only be reduced to a limited extent owing to the solubility of the intermediates. The practical Li/S energy density (*E*_p_, W h kg^−1^) was calculated using eqn (1):1
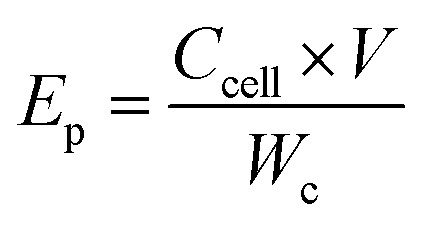
where *W*_c_ (kg) is the weight of the cell components (cathode, anode, separator, and electrolyte), *V* (V) is the potential, and *C*_cell_ (A h) is the discharge capacity of the cell. When all the components were considered, the Li/SP450 cell offered a high practical density of 250 W h kg^−1^ because it did not contain any inactive components and the amount of electrolyte was reduced by using a carbonate-based electrolyte. Specifically, 3.7 μL of electrolyte was used per 1 mg of sulfur, which is similar to the previously reported minimum electrolyte amount. However, the sulfur utilization was two times higher than that in previous reports at similar current densities (75% and 35%, respectively). Previous studies on the use of SPAN have focused on the cycle performance rather than the electrolyte amount. Typically, for SPAN electrodes, 60 μL of electrolyte has been used per 1 mg of sulfur. If a SP450-based cell was constructed using the same products, such as lithium foil and a separator, the capacity would be 96 W h kg^−1^, despite SP450 having a higher reversible gravimetric capacity than all reported SPAN-based electrodes. Thus, SP450 exhibited a high practical specific energy density because the free-standing electrode was composed of only active components and a small amount of electrolyte.

**Fig. 5:**
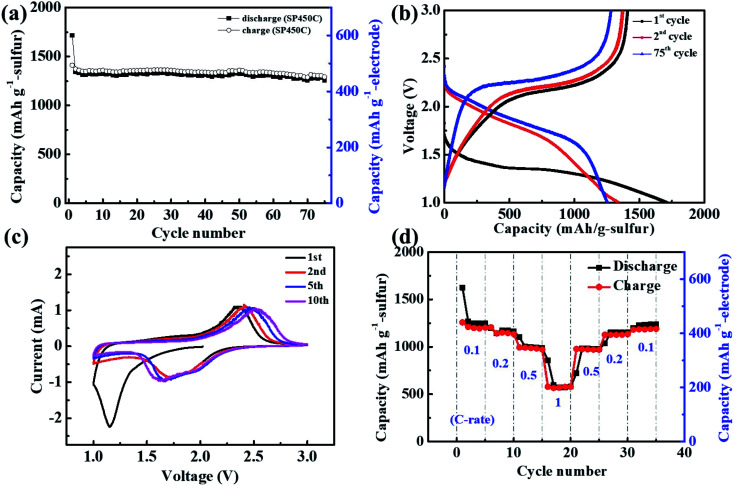
(a) Cyclability, (b) charge–discharge curves of the Li/SP450-C cell at current density of 100 mA g^−1^. (c) Cyclic voltammograms at 0.1 mV s^−1^, and (d) rate capability of the Li/SP450-C cell.

To improve the cell performance, we introduced a small amount of conducting agent (10 wt% MWCNTs) into the electrode because fast electron transfer was difficult in the thick SPAN electrode. The obtained SP450-C electrode had a sulfur content of 35 wt%. As shown in Fig. 5a, the Li/SP450-C cell exhibited stable cyclability and maintained a discharge capacity of 440 mA h g_electrode_^−1^ (1258 mA h g_sulfur_^−1^) at the 75^th^ cycle. Fig. 5b shows the changes in the charge–discharge curves of SP450-C during cycling. The discharge curve had one plateau, the potential of which increased after the 2^nd^ cycle. The average operating potential of SP450-C was higher than that of SP450, which may be due to the addition of conducting agents. The Li/SP450-C cell exhibited an initial discharge capacity of 600 mA h g_electrode_^−1^ (1715 mA h g_sulfur_^−1^), which decreased to 470 mA h g_electrode_^−1^ (1343 mA h g_sulfur_^−1^) at the 1^st^ charge. Irreversible behavior was also observed, despite the addition of the conducting agents. Both conducting agents and residual Li increase the electrical conductivity of SPAN, although their effects are different.

Fig. 5c shows cyclic voltammograms of the SP450-C cathode at a scan rate of 0.1 mV s^−1^. The basic electrochemical characteristics of the cyclic voltammograms are similar to those of the charge–discharge curve profiles. In the 1^st^ cycle, SP450-C exhibited one cathodic peak at 1.2 V. However, in subsequent scans, the onset potential of this peak shifted to a more positive value with broad peaks observed at 1.7 and 2.1 V. These broad peaks were reversible, corresponding to the formation of Li_2_S from free sulfur after cleavage of C–S and S–S bonds. In addition, the reduction peak initially appeared at 2.4 V and then shifted toward higher potentials during cycling, which suggests that the electrical conductivity improved during cycling, as shown in Fig. 4e. Fig. 5d shows the rate capability of the Li/SP450-C cell as the current density was gradually increased from 0.1 to 1 C. The SP450-C cathode exhibited rate capabilities of 1266, 1142, 1001, and 598 mA h g_sulfur_^−1^ at current rates of 0.1, 0.2, 0.5, and 1 C, respectively, which are high values considering the absence of a current collector. This reports shows higher utilization of sulfur with low E/S ratio than previous reports, which shown in Table S2.† These results show the great potential of bulk-type SPAN disk electrodes with carbonate-based electrolytes for realizing Li/S batteries with high energy densities and long cyclability for future energy storage applications.

## 4. Conclusions

A free-standing sulfur cathode was prepared by a one-step process, in which compacted sulfur and PAN powders were heated to 450 °C. The SP450 cathode consisted of SPAN without any inactive components, such as a conducting agent, binder, or current collector. The electronic conductivity of SPAN increased more than 15 000 times following lithiation, which may be related to the absence of a conducting agent in SP450. In addition, the SP450 cathode had a high areal loading of 16.37 mg cm^−2^ and exhibited a high discharge capacity of 674 mA h g_electrode_^−1^ with a reversible capacity of 517 mA h g_electrode_^−1^ (1260 mA h g_sulfur_^−1^) after the 10^th^ cycle. Considering the weight of the anode, separator, cathode, and electrolyte without packaging, the corresponding Li/S cell exhibited a high practical energy density of 250 W h kg^−1^ because of the small amount of electrolyte (3.7 mL g_sulfur_^−1^) and the high gravimetric capacity of SP450. However, to achieve commercialization, the power density of the Li/SP450 cell should be further improved.

## Conflicts of interest

There are no conflicts to declare.

## Supplementary Material

RA-011-D1RA02462K-s001
