# Supplementary material for: Binder-free and high-loading sulfurized polyacrylonitrile cathode for lithium/sulfur batteries
Source: RSC Adv. 2021 Apr 30;11(26):16122–30. doi: 10.1039/d1ra02462k (PMC9030391; doi:10.1039/d1ra02462k)
Supplement: RA-011-D1RA02462K-s001 [file RA-011-D1RA02462K-s001.pdf]

Electronic Supplementary Material (ESI) for RSC Advances.
This journal is © The Royal Society of Chemistry 2021

Electronic Supporting Information

Binder-free and high-loading sulfurized polyacrylonitrile cathode for lithium/sulfur batteries

Huihun Kim, Changhyeon Kim, Milan K. Sadan, Hyewon Yeo, Kwon-Koo Cho, Ki-Won Kim, Jou-Hyeon Ahn and Hyo-Jun Ahn*

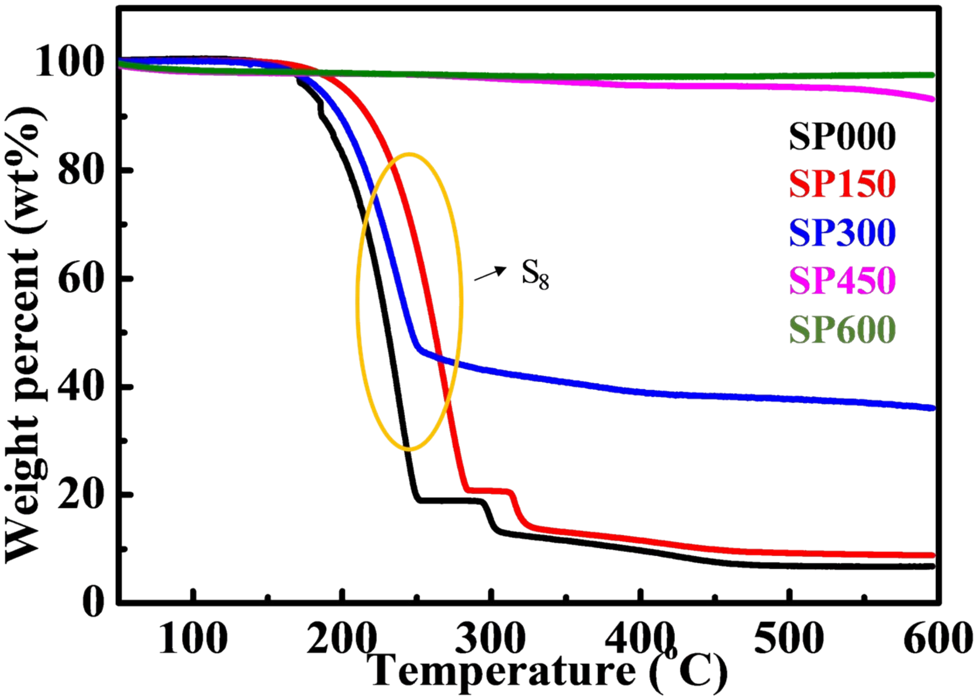

Fig. S1. TGA curves of SPpellet samples.

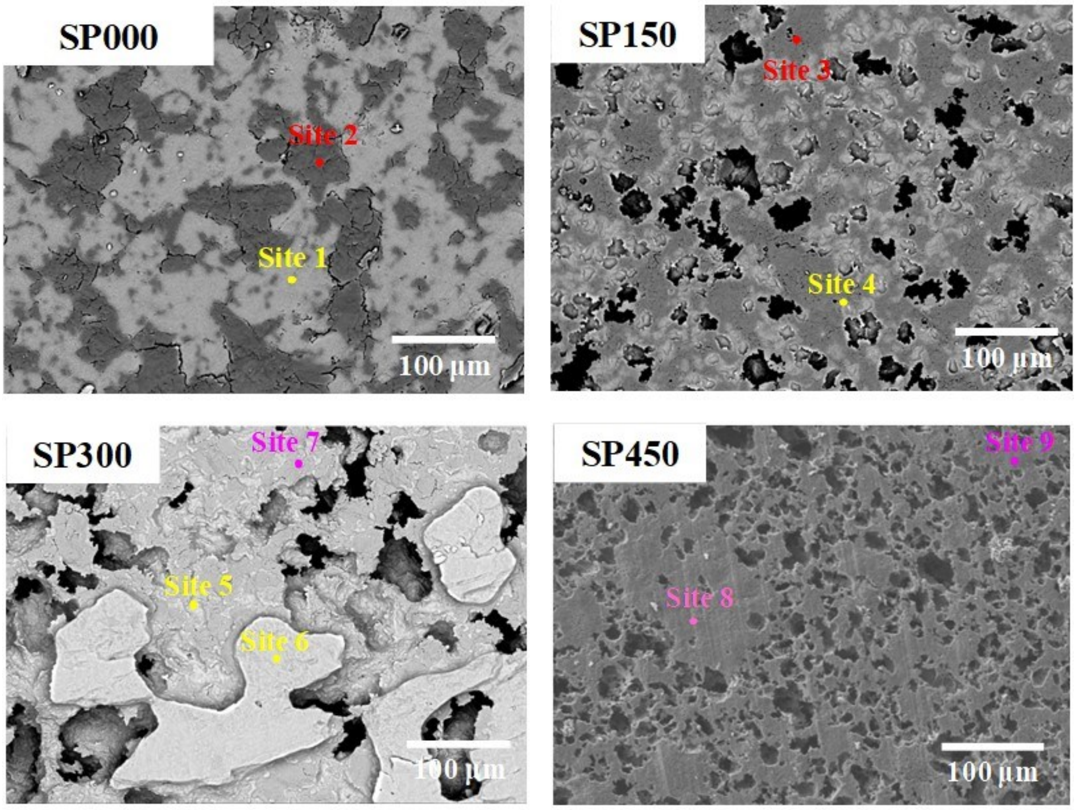

Unit : at%

Sample/position		N	C	S
SP000	Site 1	-	-	100
	Site 2	23.37	76.63	-
SP150	Site 3	-	-	100
	Site 4	21.45	78.55	-
SP300	Site 5	-	-	100
	Site 6	-	-	100
	Site7	6.21	72.7	21.5
SP450	Site 8	14.9	65.8	19.3
	Site 9	14.7	64.5	20.8

Fig. S2. SEM images and EDS point mapping results for SPAN electrodes

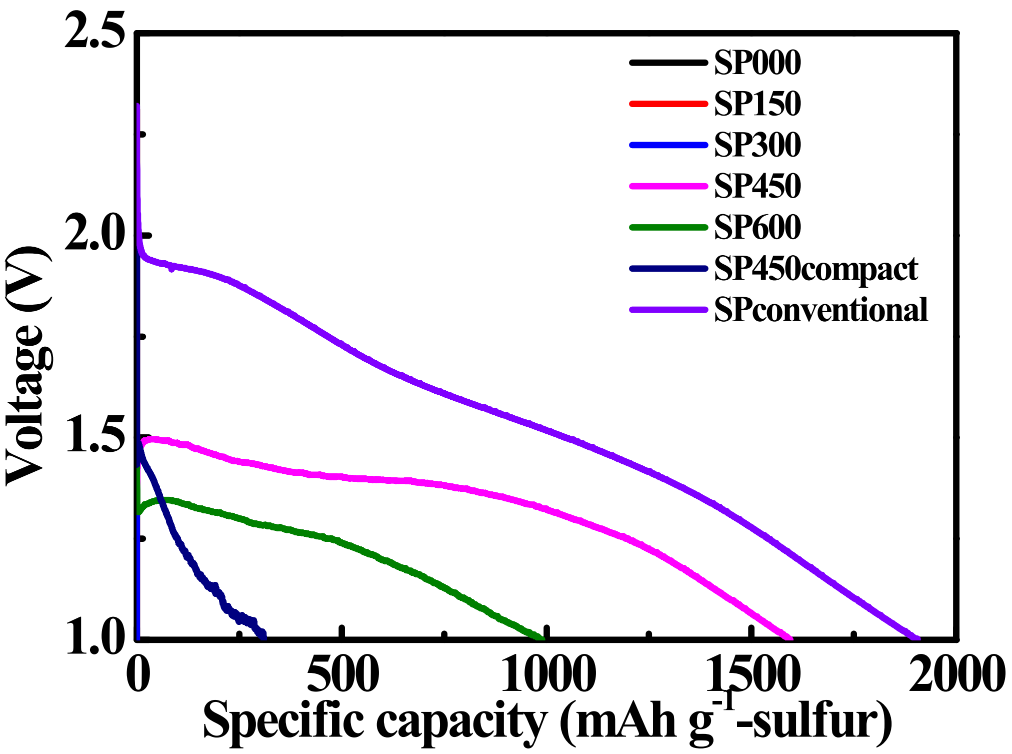

Fig. S3. Discharge–charge curves of various Li/SPAN cells at current density of 15 mA g^{-1}.

elecrode·

Table S1. Components of Li/SPAN cells.

Sample name	Cathode				Current collector	Electrolyte	Separator	Anode	Capacity	Capacity	Capacity
	Active material (SP450)	Binder (β – cyclodextrin)	conducting agent (MWCNT)	Total cathode	Al foil	1 M $LiPF_6$ + EC/DEC	Celgard 2400	Lithium foil	Based on sulfur	Based on electrode without current collector	Based on electrode with current collector
SP450compact	24.1 mg	-	-	24.1 mg	-	30 mg	2.1 mg	12.6 mg	321 mAh g^{-1}	132 mAh g^{-1}	132 mAh g^{-1}
SP450conventional	0.776 mg	0.097 mg	0.097 mg	0.97 mg	3.83 mg	10 mg	2.1 mg	12.6 mg	1931 mAh g^{-1}	649 mAh g^{-1}	131 mAh g^{-1}
SP450	16.37 mg	-	-	16.37 mg	-	30 mg	2.1 mg	12.6 mg	1656 mAh g^{-1}	679 mAh g^{-1}	679 mAh g^{-1}

Table S2. Comparison of previous high loading sulfur with this report.

Sulfur loading mg cm^{-2}	E/S ratio uL/mg	Reversible capacity mAh g^{-1}-sulfur (mAh cm^{-2})	Ref.
10.2	20	600	1
8	15	1000 (8)	2
13.2	15	791 (7)	3
9.1	9	400	4
10	5.7	1000	5
13.5	3	600	6
6.87	3.7	1230	This work

E/S ratio: Electrolyte/sulfur ratio

Reference

1 C. Chen, J. Jiang, W. He, W. Lei, Q. Hao and X. Zhang, *Adv. Funct. Mater.*, 2020, **30**, 1909469.

2 J. Shi, Q. Kang, Y. Mi and Q. Xiao, *Electrochim. Acta*, 2019, **324**, 134849.

3 J. Wang, G. Yang, J. Chen, Y. Liu, Y. Wang, C.-Y. Lao, K. Xi, D. Yang, C.J. Harris, W. Yan, S. Ding and R.V. Kumar, *Adv. Energy Mater.*, 2019, **9**, 1902001.

4 J. Liao, J. Wang, Z. Liu and Z. Ye, *ACS Appl. Energy Mater.*, 2019, **2**, 6732-6740.

5 X. Yang, X. Gao, Q. Sun, S.P. Jand, Y. Yu, Y. Zhao, X. Li, K. Adair, L.-Y. Kuo, J. Rohrer, J. Liang, X. Lin, M.N. Banis, Y. Hu, H. Zhang, X. Li, R. Li, H. Zhang, P. Kaghazchi, T.-K. Sham and X. Sun, *Adv. Mater.*, 2019, **31**, 1901220

6 G. Li, W. Lei, D. Luo, Y. Deng, Z. Deng, D. Wang, A. Yu and Z .Chen, *Energy Environ. Sci.*, 2018, **11**, 2372-2381.
